# The Success of Newborn Screening Beyond War: An International Collaborative Case of Purine Nucleoside Phosphorylase (PNP) Deficiency

**DOI:** 10.3390/ijns11030079

**Published:** 2025-09-16

**Authors:** Alessandra Bettiol, Roberta Damiano, Nataliia Mytsyk, Nataliia Samonenko, Gabriella Cericola, Carsten Speckmann, Nataliia Olkhovich, Renzo Guerrini, Giancarlo la Marca

**Affiliations:** 1 Department of Experimental and Clinical Biomedical Sciences “Mario Serio”, University of Florence, 50134 Florence, Italy; alessandra.bettiol@unifi.it; 2 Newborn Screening, Clinical Biochemistry and Clinical Pharmacy Laboratory, Department of Neuroscience and Medical Genetics, Meyer Children’s Hospital IRCCS, 50139 Florence, Italy; roberta.damiano@meyer.it; 3 National Specialized Children’s Hospital “Ohmatdyt”, 01135 Kyiv, Ukrainenatalisam@gmail.com (N.S.);; 4 Institute for Immunodeficiency, Center for Chronic Immunodeficiency, Faculty of Medicine, Medical Center, University of Freiburg, 79106 Freiburg, Germany; gabriella.cericola@uniklinik-freiburg.de (G.C.);; 5 Division of Pediatric Hematology and Oncology, Department of Pediatric and Adolescent Medicine, University Medical Center Freiburg, University of Freiburg, 79110 Freiburg, Germany; 6 Department of Neuroscience and Medical Genetics, Meyer Children’s Hospital IRCCS, 50139 Florence, Italy; 7 Department of Neuroscience, Pharmacology and Child Health (NEUROFARBA), University of Florence, 50139 Florence, Italy

**Keywords:** newborn screening, purine nucleoside phosphorylase deficiency, severe combined immunodeficiency, war, public health, tandem mass spectrometry, dried blood spot

## Abstract

Ukraine’s healthcare system has shown remarkable resilience in continuing newborn screening (NBS), beyond the challenges of war. Amid the conflict, a Ukrainian newborn screened positive for an extremely rare severe combined immunodeficiency (SCID)–purine nucleoside phosphorylase (PNP) deficiency. Ukraine successfully carried out NBS on a neonatal dried blood spot (DBS) by real-time PCR, which showed remarkably reduced T-cell receptor and kappa-deleting recombination excision circles (TREC/KREC). Retesting was delayed due to communication difficulties with the family. Whole exome sequencing on a new DBS confirmed the diagnosis. The newborn was a candidate for allogeneic hematopoietic stem cell transplantation (HSCT), the only curative treatment. HSCT is a complex procedure still ongoing in Ukraine despite the conflict. However, due to the psychosocial strain, the family sought medical support in Germany, where HSCT was performed successfully at 6 months. As part of a collaborative initiative with Italy, PNP biomarkers were quantified on the same DBSs using tandem mass spectrometry, according to the protocols established for SCID NBS in Tuscany, serving as a proof of concept of its diagnostic performance. This case highlights the importance of sustaining preventive and life-saving healthcare services, and reflects the key role of international partnerships in upholding the right to healthcare in times of crisis.

## 1. Introduction

Ukraine’s healthcare system has shown remarkable resilience in continuing preventive health programs, such as newborn screening (NBS) for inherited metabolism and immunity disorders, despite the difficulties of the ongoing nation-wide armed conflict. In this paper, we report the case of a Ukrainian male newborn, born amid the war and screened positive at NBS for an extremely rare severe combined immunodeficiency (SCID)–purine nucleoside phosphorylase (PNP) deficiency.

PNP deficiency is an extremely rare autosomal recessive disease; its estimated incidence is 1 in 50,000–100,000 live births, and it accounts for around 4% of all cases of SCID [1,2]. The disease is due to loss-of-function mutations in the *PNP* gene located at chromosome 14q11.2, encoding a key enzyme catalyzing the reversible phosphorolysis of guanosine (Guo), deoxyguanosine (dGuo), inosine (Ino), and deoxyinosine (dIno), and to their respective purine bases and pentose-1-phosphates. In the presence of a defective PNP function, its substrates undergo alternative metabolic pathways, with the accumulation of toxic metabolites. Among them, intracellular accumulation of deoxyguanosine triphosphate (dGTP) derived from dGuo can interfere with DNA synthesis and repair, thus affecting lymphocyte maturation and resulting in a progressive immunodeficiency. The genetic heterogeneity in terms of mutations in the *PNP* gene results in various levels of residual enzyme activity, determining a wide spectrum of clinical phenotypes ranging from mild adult-onset forms to neonatal-onset SCID [3].

The clinical suspicion of PNP deficiency arises in the presence of recurrent infections caused by common pathogens or opportunistic organisms; also, neurological abnormalities and autoimmunity can occur in up to two-thirds and one-third of patients, respectively, with hematologic malignancies and developmental delay occurring in some cases [4]. From a laboratory point of view, the disease is characterized by T-lymphopenia, varied B-cell abnormalities, and low serum uric acid [5]. Supportive treatments to reduce the risk of infections include immunoglobulin replacement therapy, antimicrobial prophylaxis (i.e., to prevent *Pneumocystis pneumonia*), and the withholding of live vaccines [6,7]. However, hematopoietic stem cell transplantation (HSCT) is considered the only curative treatment for the underlying immunodeficiency [7]. Early HSCT intervention, ideally within the first months of life, is crucial to promptly restore PNP activity, thus allowing for immune cell proliferation and metabolic detoxification before irreversible damage occurs. Early detection of PNP deficiency by NBS programs is therefore crucial to promptly consider HSCT and avoid severe complications [8,9].

Ukraine has an established NBS for SCID, based on the measurement of T-cell receptor excision circle (TREC) and kappa-deleting recombination excision circle (KREC) levels by quantitative real-time PCR on neonatal dried blood spots (DBSs) [10]. The newborn described in this case report was correctly identified as screen positive for SCID at NBS based on TREC/KREC analysis. However, following the confirmed diagnosis, the family sought medical help in Germany, looking for a safer place to live and care for their child. Biochemical monitoring of markers of PNP deficiency in the pre- and post-HSCT phase was further performed in collaboration with Tuscany (Italy).

**Figure 1:**
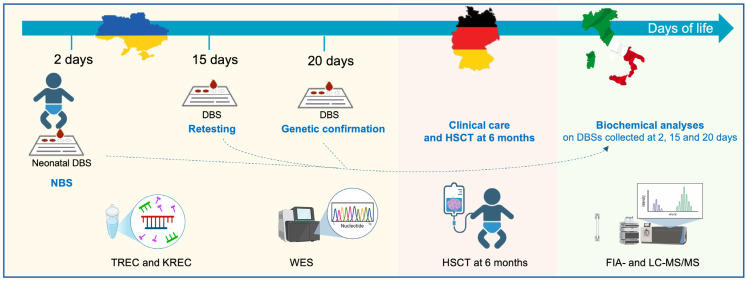
Graphical representation of the clinical and analytical workflow in the framework of an international partnership between Ukraine, Germany, and Italy.

In Tuscany, first-tier tests for NBS of SCID are based on the quantification of Guo, dGuo, Ino, and dIno metabolite levels by flow injection analysis–tandem mass spectrometry (FIA-MS/MS), and second-tier tests for biochemical confirmatory purposes are performed by liquid chromatography–tandem mass spectrometry (LC-MS/MS) [11,12,13,14]. Following the implementation of PNP SCID in NBS programs in Tuscany in 2014, no new cases of PNP deficiency have been identified from approximately 250,000 tests, likely due to the rarity of the disease. In the framework of this international partnership (Figure 1), the neonatal DBS sample of this patient was sent from Ukraine to Tuscany and re-analyzed by MS/MS, as a proof-of-concept analysis to confirm the feasibility of MS/MS-based NBS for the detection of this rare SCID.

## 2. Case Presentation

A male Ukrainian child was born at term in good general condition. NBS from DBS was performed according to routine protocols at 2 days of life [10]. Shortly after birth, he also received Bacillus Calmette–Guérin (BCG) vaccination, a live-attenuated vaccine to prevent mycobacterial infections, according to standard vaccination programs in Ukraine. The result from the NBS (available at 5 days) showed remarkably reduced TREC and KREC copy levels (TREC 62 copies; KREC 23 copies; cut-off threshold at time of patient’s NBS >2000 copies per 10^6^ cells; currently revised to >5395 copies per 10^6^ cells for TREC and >1312 copies per 10^6^ cells for KREC), compatible with a SCID profile (Table 1).

According to standard NBS protocols, screen positive cases should undergo retesting on a new DBS sample. In this case, retesting was delayed due to communication difficulties with the parents and was finally performed at 15 days (with results available at 19 days), confirming the profile (TREC 398 copies; KREC 17 copies). Also, flow cytometry analysis identified the typical phenotype with reduced T and B cells, suggestive of SCID. Genetic test by whole exome sequencing (WES) on a new DBS (collected at 20 days) confirmed the diagnosis of PNP deficiency [c.751delA (p.Ser251Alafs*11)+/+], which was finally made at 40 days of life. The family was counseled about the diagnosis and preventive measures to protect the child from early infections. Live-attenuated vaccines (such as BCG) should be withheld in these patients; however, the newborn had been vaccinated immediately after birth, according to routine practice, before NBS results were available. The patient started supportive prophylactic treatments to reduce the risk of infections, including immunoglobulin replacement therapy, and antimicrobial prophylaxis against *Pneumocystis pneumonia*. The child was a candidate for allogenic (allo) HSCT with chemotherapy-based conditioning, as the first-line curative therapeutic option. Allo HSCT is a complex procedure that is still ongoing in Ukraine despite the conflict. However, the family opted to seek clinical help in Germany. The patient was admitted to the University Hospital of Freiburg (Germany) at 11 weeks of age. He was in good medical condition and was continuously free from infections including the absence of BCG vaccine-related complications. Flow cytometry analysis confirmed the phenotype of severely reduced T and B cells, including very low naïve T cells. Allogenic HSCT from an HLA-matched unrelated donor was finally successfully performed at 6 months of age.

The University Hospital of Freiburg has a longtime established relationship with the Laboratory of Newborn Screening, Clinical Biochemistry and Clinical Pharmacy (Meyer Children Hospital IRCCS, Florence, Italy) for the quantification of biochemical markers of SCID for diagnostic and follow-up purposes.

**Figure 2:**
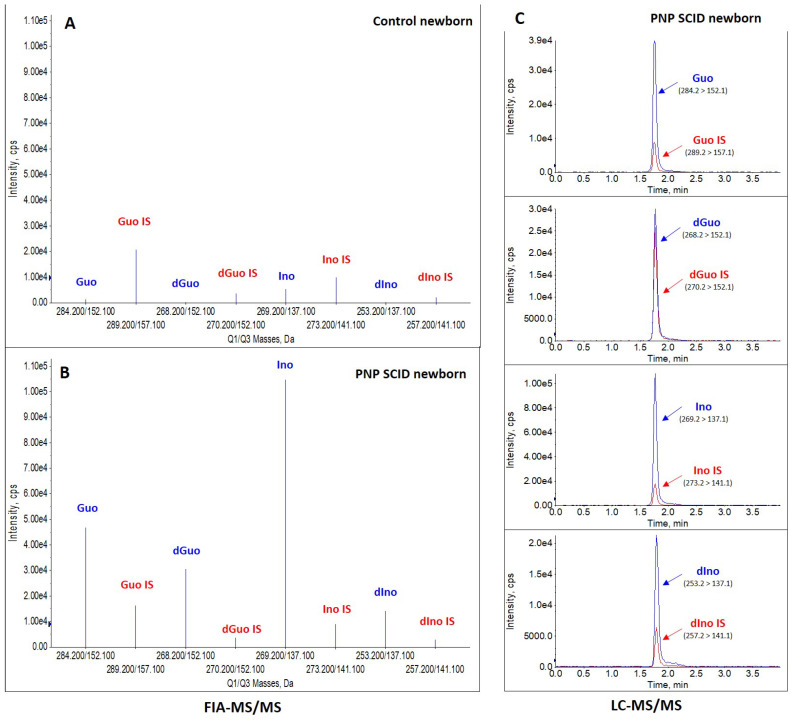
(**A,B**) FIA-MS/MS and (**C**) LC-MS/MS experiments show increased concentrations of Guo, dGuo, Ino, and dIno in the neonatal DBS sample (collected at 2 days of life) of the patient with PNP SCID (**B,C**) as compared to a control newborn (**A**). FIA: flow injection analysis; IS: Internal Standard; LC: liquid chromatography; MS/MS: tandem mass spectrometry; PNP: purine nucleoside phosphorylase; SCID: severe combined immunodeficiency.

Quantification of PNP biomarkers Guo, dGuo, Ino, and dIno was performed on the same DBS samples used for TREC/KREC analysis (collected at 2, 15, and 20 days of life), for biochemical confirmation of the profile. After obtaining ad hoc written informed parental consent, we contacted the National Newborn Screening Laboratory in Ukraine, asking to retrieve and send the stored neonatal DBS that had been collected at the patient’s birth (2 days of life). Moreover, DBS collected at 15 and 20 days of life were also retrieved and sent to the Meyer children Hospital IRCSS (Florence). The DBS samples were prepared as previously described [11,12], and both a first-tier test by FIA-MS/MS and second-tier test by LC-MS/MS were performed according to the published methods [11,12]. Apart from PNP biomarkers, and adenosine (Ado) and 2-deoxyadenosine (dAdo), biomarkers of adenosine deaminase (ADA) deficiency were quantified [11] due to the similarity between the two syndromes. High levels of purine metabolites Guo, dGuo, Ino, and dIno were found in all three DBS samples collected at different times, as shown in Table 1 and Figure 2 (for the neonatal DBS). Particularly, analyses performed on neonatal DBSs indicated that the patient would have been classified as screen positive if NBS for PNP deficiency by FIA- and LC-MS/MS was performed.

## 3. Discussion

This case report underscores the importance of continuing routine preventive health programs, such as NBS, despite critical geo-political scenarios. In the last 2.5 years, more than 445,000 newborns underwent NBS in Ukraine; 97 were screen positive for SCID at the first-tier test and 32 of them were screen positive after retesting. Overall, nine cases of genetically confirmed SCID have been identified. The successful story reported in this manuscript confirms the key role of NBS for pre-symptomatic diagnosis of PNP deficiency and early therapeutic intervention. Positive screening has a well-known burden on parental well-being and psychosocial functioning, irrespective of social setting [15,16,17]. We can well imagine how this emotional impact is amplified in families already burdened by a war conflict, as confirmed by the difficulties of initially approaching this family for retesting and confirmatory analyses, which further highlights the need to improve multidisciplinary communication strategies. The exodus lived by this family underlines the humanitarian crisis of war and emphasizes the vital importance of international partnerships between healthcare organizations. Concomitantly, this case offered the opportunity to conduct a proof-of-concept analysis, confirming the diagnostic performance of MS/MS-based NBS for the detection of PNP deficiency on neonatal DBS.

## Figures and Tables

**Table 1:** Molecular analysis of TREC and KREC levels, genetic testing, and quantification of purine biomarkers of PNP deficiency by MS-MS in the DBS samples collected at 2, 15, and 20 days of life.

	Neonatal DBS (2 Days)	1st Retest (15 Days)	2nd Retest (20 Days)
**TREC ^§^** (considered cut-off threshold > 2000 copies per 10^6^ cells, currently revised to >5395 copies per 10^6^ cells)	62	398	**-**
**KREC ^§^** (considered cut-off threshold > 2000 copies per 10^6^ cells, currently revised to >1312 copies per 10^6^ cells)	23	17	**-**
**Whole exome sequencing (WES) ^§^**	**-**	**-**	c.751delA (p.Ser251Alafs*11)+/+
First-tier test by FIA-MS/MS *			
Guo (n.v. < 4.9 μmol/L)	16.9	8.8	3.3
dGuo (n.v. < 0.6 μmol/L)	14.1	6.1	4.7
Ino (n.v. < 61.5 μmol/L)	67.9	66.8	97.7
dIno (n.v. < 7.7 μmol/L)	29.0	97.7	8.9
Second-tier test by LC-MS/MS *			
Guo (n.v. < 1.1 μmol/L)	46.4	33.8	10.2
dGuo (n.v. 0 μmol/L)	12.5	6.7	4.3
Ino (n.v. < 16.8 μmol/L)	58.8	61.0	91.8
dIno (n.v. < 0.1 μmol/L)	36.5	14.5	10.1

^§^ Performed at the National Specialized Children’s Hospital “Ohmatdyt”, Kyiv, Ukraine. * Performed at the Meyer Children Hospital IRCCS, Florence, Italy. ADA SCID biomarkers (Ado and dAdo) were within normal range in FIA-MS/MS and LC-MS/MS.

## Data Availability

All data related to this case report are available in the main text of this paper.

## Abbreviations

The following abbreviations are used in this manuscript:

NBS	Newborn screening
SCID	Severe combined immunodeficiency
PNP	Purine nucleoside phosphorylase
Guo	Guanosine
dGuo	Deoxyguanosine
Ino	Inosine
dIno	Deoxyinosine
dGTP	Deoxyguanosine triphosphate
HSCT	Hematopoietic stem cell transplantation
TREC	T-cell receptor excision circles
KREC	Kappa-deleting recombination excision circles
DBS	Dried blood spot
FIA	Flow injection analysis
MS/MS	Tandem mass spectrometry
LC	Liquid chromatography
BCG	Bacillus Calmette–Guérin
WES	Whole exome sequencing
Allo	Allogenic
Ado	Adenosine
dAdo	2-deoxyadenosine
ADA	Adenosine deaminase
